# The Anatomo-Electrical Network Underlying Hypermotor Seizures

**DOI:** 10.3389/fneur.2018.00243

**Published:** 2018-04-11

**Authors:** Xiu Wang, Wenhan Hu, Kai Zhang, Xiaoqiu Shao, Yanshan Ma, Lin Sang, Zhong Zheng, Chao Zhang, Junjv Li, Jian-guo Zhang

**Affiliations:** ^1^Department of Neurosurgery, Beijing Tian Tan Hospital, Capital Medical University, Beijing, China; ^2^Stereotactic and Functional Neurosurgery Laboratory, Beijing Neurosurgical Institute, Capital Medical University, Beijing, China; ^3^Beijing Key Laboratory of Neurostimulation, Beijing, China; ^4^Department of Neurology, Beijing Tian Tan Hospital, Capital Medical University, Beijing, China; ^5^Epilepsy Center, Medical Alliance of Beijing Tian Tan Hospital, Peking University First Hospital Fengtai Hospital, Beijing, China; ^6^Department of Neurosurgery, Hainan General Hospital, Haikou, China

**Keywords:** semiology, hypermotor, interictal ^18^FDG-PET, stereo-electroencephalography, network, cingulate cortex

## Abstract

**Purpose:**

Hypermotor seizures (HMS) can be triggered by different epileptogenic foci and thus common symptomatic networks generating HMS may exist among these patients. The goal of the present study was to investigate the specialized networks underlying HMS by analyzing interictal ^18^FDG-PET imaging and ictal stereo-electroencephalography (SEEG) recordings.

**Methods:**

Fourteen patients with HMS were retrospectively analyzed. HMS were classified into HMS1 and HMS2 according to the speed and intensity of the motor seizure behavior. Then, the interictal PET data of patients was compared with those of 18 healthy controls using statistical parametric mapping to identify regions with significant hypometabolism. Ictal SEEG recordings were reviewed to identify the spreading areas at the beginning of HMS occurrence.

**Results:**

Compared to controls, patients with HMS presented significant hypometabolism in the bilateral anterosuperior insular lobes, mesial premotor cortex (MPMC), middle cingulate cortex (MCC), as well as in the bilateral caudate nuclei. When comparing patients in the two HMS subgroups with controls, more extensive hypometabolic areas were seen in HMS1 patients than in HMS2 patients, including the orbitofrontal cortex (OFC), the temporal pole, and the anterior cingulate cortex (ACC). OFC and ventromedial prefrontal cortex was also found significantly hypometabolic in patients with HMS1 when compared with HMS2 directly. SEEG recordings further suggested that insula, MCC, and MPMC were commonly recruited at the beginning of HMS.

**Conclusion:**

We have identified a specialized interictal hypometabolic pattern in patients with HMS. A network involving the anterosuperior insula, mesiofrontal cortex (MCC-MPMC), and caudate nucleus may contribute to the generation of HMS. ACC, OFC, and temporal pole are possibly associated with the affective components of HMS. Our findings provide further insight into understanding the network basis of HMS semiology.

## Introduction

Hypermotor seizures (HMS) are primarily characterized by complex behavior involving proximal segments of the limbs and trunk, producing pedaling, kicking, pelvic thrusting or rocking movements (1). This seizure behavior can be triggered by epileptogenic foci in the frontal lobe (2), the temporal lobe (3), or the insular lobe (4). A common network, including cortical and subcortical structures (1, 5), may be involved in the generation of HMS. Ictal stereo-electroencephalography (SEEG) recordings (2) suggested that mesiofrontal areas, including ventromedial frontal cortex and mesial premotor cortex (MPMC), were primarily associated with HMS onset. An ictal perfusion study using single photon emission computed tomography (SPECT) further concluded bilateral mesiofrontal regions, caudate nuclei and temporal lobe structures were involved the generation of HMS (6).

Compared to ictal SPECT, interictal positron emission tomography with ^18^F-flurodexyglucose (^18^FDG-PET) is more widely used in the presurgical evaluation of epilepsy patients. The topography of interictal hypometabolism was found be related to brain areas which generate the clinical semiology (7–9) of ictal onset and spread (10), and thus provides guidance for the design of SEEG implantation. Accordingly, the first goal of the present study was to confirm the specialized hypometabolic patterns in patients with HMS. Besides, two types of HMS can be distinguished according to motor speed and intensity. HMS1 primarily consists of motor behavior with marked agitation and a facial expression of fear. HMS2 consists of mild agitation and is usually associated with tonic posturing. Therefore, PET scans in subgroups were further analyzed to investigate the difference of hypometabolic patterns between HMS1 and HMS2. These results may allow a deeper understanding of HMS and the similarities and differences between HMS1 and HMS2.

## Materials and Methods

### Patient Selection

Fourteen consecutive patients with refractory epilepsy, HMS behavior (occurring at the beginning or during seizure episode) and no history of cranial surgery were retrospectively recruited between 2015 and 2016. Seizure manifestations of video electroencephalography (EEG) were reviewed (83 HMS in total), and HMS were classified according to the following criteria: HMS1 consisted of body rocking, kicking, or boxing behavior with marked agitation, often with a fearful or anxious expression; HMS2 consisted of horizontal movements or rotations of the trunk and pelvis, or pelvic thrusts while lying on the bed (2); patients with sitting up or standing up behaviors without marked agitation were included in HMS2. Xiu Wang and Kai Zhang reviewed the ictal semiology independently, and disagreements were resolved through discussion with a senior epileptologist (Xiaoqiu Shao). We selected ictal symptoms proven to be habitual and reproducible when patient presented some different HMS behaviors between seizure episodes. Eighteen healthy volunteers of similar age were included in this study (male: *n* = 10, age: 22.6 ± 3.3 years). These controls were free of neurological or psychiatric disorders and their MRI scans were normal. Informed consent with protocols approved by the Institutional Review Boards of the Beijing Tiantan Hospital were obtained from all included members, including patients and volunteers. The study has been performed in accordance with the ethical standards laid down in the 1964 Declaration of Helsinki and its later amendments.

### ^18^FDG PET Data Acquisition

PET scans of all patients were obtained in the interictal state with the same protocols as healthy subjects. The ^18^FDG-PET examinations were performed under standard resting conditions using the GE Discovery ST PET-CT system (300 mm FOV, matrix 192 × 192, 3.27 mm slice thickness). The ^18^FDG was injected *via* IV at a mean dose of 310 MBq/70 kg body weight. The reconstructed images were corrected for attenuation using transmission scans obtained from a germanium source. PET scans of all patients were obtained within 6 months before epilepsy surgery evaluation. No patients had clinical seizures less than 6 h before or during the PET scan.

### PET Image Processing and Statistical Parametric Mapping (SPM) Analysis

The site of the epileptogenic focus was confirmed by scalp-EEG, SEEG, and surgery information, and we transposed the horizontal PET images of patients with right epileptogenic foci using Grocer (V2.15, Senhua Zhu, UPenn Med., 2011) as applied in previous study (6). Therefore, all the ictal onsets were lateralized to the left for analysis. Whole-brain statistical analysis was performed at the voxel-level using SPM8 software (Wellcome Department of Cognitive Neurology, University College, London, UK). The PET images were spatially normalized onto the Montreal Neurological Institute atlas (voxel size: 2 mm × 2 mm × 2 mm). The images were then smoothed with a Gaussian filter (8 mm FWHM) to increase the signal-to-noise ratio. The resulting PET images were divided by the individual mean FDG uptake value of global brain to control for individual variations. The parametric images of the whole HMS group and HMS subgroups were, respectively, compared with those of a group of 18 healthy subjects using voxel-based independent *t*-test analysis, as implemented in SPM8 software. Then, brain metabolism was directly compared between patients with HMS1 and patients with HMS2. A cluster threshold of 100 voxels was applied, with no correction for multiple comparisons (*p* < 0.001) (11).

### SEEG Evaluation and Surgery Resection

Stereo-electroencephalography electrodes were implanted when non-invasive evaluation could not precisely localize the seizure onset zone. Long-term recordings were performed after implantation using a NIHON KOHDEN video-EEG monitoring system. Electroclinical features of habitual seizures were recorded in all patients. The ictal onset zone and the spreading areas recruited at the beginning of HMS were reviewed by Zhong Zheng, who was blinded to the patients’ information and PET analysis results. Ictal onset zone is defined as the cortex area with the first clear ictal SEEG change prior to or concurrent with the earliest clinically detectable ictal sign (2). The resection area was decided by multidisciplinary case seminar according to non-invasive evaluation data and SEEG recordings.

**Figure 1:**
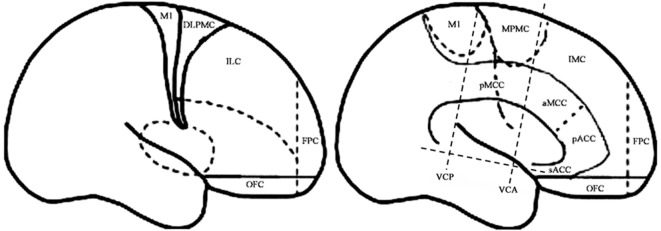
Schematic anatomical division of the frontal lobe used for description in the present study [modified from Bancaud and Talairach (14) and Rheims et al. (2)]. M1, primary motor cortex; DLPMC, dorsolateral premotor cortex; FPC, frontopolar cortex; ILC, intermediate dorsolateral frontal cortex; IMC, intermediate mesial cortex; MPMC, mesial premotor cortex; OFC, orbitofrontal cortex; sACC, subgenual anterior cingulate cortex; pACC, pregenual anterior cingulate cortex; aMCC, anterior middle cingulate cortex; pMCC, posterior middle cingulate cortex; VCA, vertical commissure anterior; VCP, vertical commissure posterior.

For the purpose of the anatomical description, the frontal lobe was divided into nine subregions proposed by Bancaud and Talairach (Figure 1), including the motor cortex [M1, Brodmann area (BA) 4], the insulo-opercular region (BAs 13, 14, 15, 16 43, 44, and 52), the dorsolateral premotor cortex (DLPMC, lateral aspects of BAs 6 and 8), the intermediate dorsolateral cortex (ILC, lateral aspects of BAs 9, 45, and 46), the MPMC (mesial aspects of BAs 6 and 8), the intermediate mesial cortex (IMC, mesial aspects of BA 9), the frontopolar cortex (BA 10), the orbitofrontal cortex (OFC, BAs 11 and 12), and the cingulate cortex. Then, the cingulate cortex in the mesial frontal lobe was further divided into two subregions according to Vogt’s study (12): the anterior cingulate cortex (ACC, BAs 32, 24a, 24b, 24cv, and 24cd) and the middle cingulate cortex (MCC, BAs 32′, 24a′, 24b′, 24c′, 24c, and 24d). The MCC was further divided into the anterior MCC (aMCC) and the posterior MCC, and the rough borderline was the vertical commissure anterior (VCA) (13).

## Results

### Clinical Data

Fourteen patients (male, *n* = 9) were included in the analysis, with a mean age of seizure onset of 7.3 ± 4.7 years old (range: 1–14 years old) and a surgery age of 20.7 ± 5.9 years old (range: 12.5–36 years old). The mean disease duration was 13.4 ± 7.8 years (range: 0.5–27 years). Eight patients underwent a total of 64 SEEG electrode implantations, with a median of 8 electrodes (range: 5–12 electrodes) implanted per patient. One patient did not receive resection surgery despite SEEG evaluation because the epileptogenic zone involved the precentral gyrus. The mean postoperative follow-up duration was 19.9 ± 7.6 months. Twelve patients received resection surgery after evaluation. Eleven patients were free from seizures (91.7%) and one patient has recurrent seizures 1 year after anterior temporal lobectomy (HMS2-7). The postoperative pathological diagnoses were the following: focal cortical dysplasia (FCD) type I (3), FCD type IIa (3), FCD IIb (4), and gliosis (2). The seizure semiology and resection areas are shown in Tables 1 and 2. HMS1 was observed in six patients and ictal fear with or without neurovegetative symptoms was the most prevalent signs (4/6). Eight patients presented with HMS2, and unilateral or bilateral tonic posture was the most prominent accompanying motor behavior (4/8).

**Table 1:** Seizure semiology and surgery resection in patients with HMS1.

	Imaging characteristics (MRI/PET-MRI coregistration)	Aura	Affective components of HMS	Motor components of HMS	Tonic signs during HMS	SEEG and/or surgery resection	Histopathology
1	MRI negative/bilateral OFC hypometabolism (R. predominant)	Uncomfortable feeling in the heart	Fearful expression	Sitting up and slight coughing, rocking back and forth, vocalization, frenetic and rapid pedaling movements of lower limbs, sometimes obscene words	–	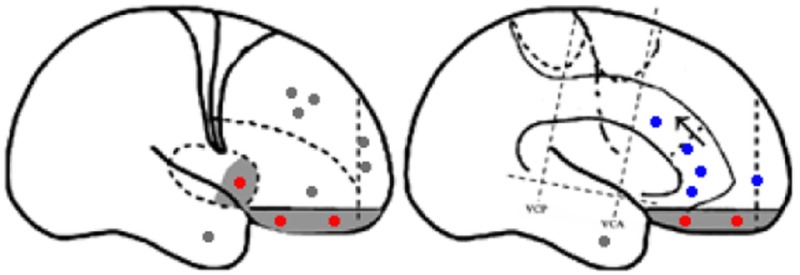	FCD IIa
2	MRI negative/R. temporal pole, R. mesial OFC hypometabolism	déjà vu/jamais vu	Angry expression	Huffing heavily and sitting up, slapping bed vigorously with bilateral hands, rapid pedaling movements of lower limbs	–	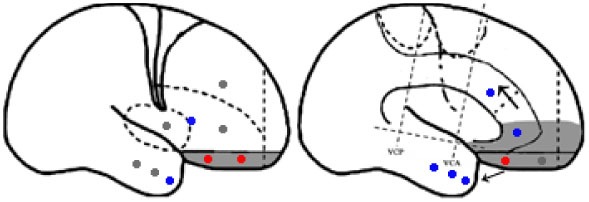	FCD Ia
3	MRI negative/L. IMC hypometabolism and subtle hypometabolism in L. superior frontal sulcus	Fear/fluster, tachycardia	Fearful expression	Vocalization, frenetic, and pedaling movements of lower limbs with large amplitude	–	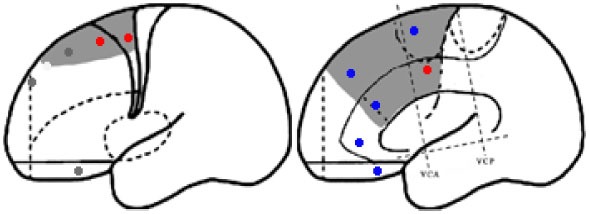	FCD IIb
4	L. temporal pole dysplasia/L. temporal lobe, insula and parietal lobe hypometabolism	déjà vu	–	Vocalization, flush, pedaling movements of lower limbs, kicking of bilateral hands	–	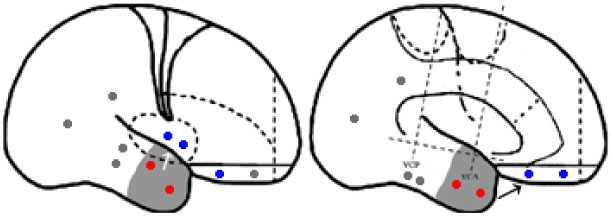	Gliosis
5	Bottom of R. superior frontal sulcus abnormality/R. superior frontal sulcus hypometabolism	Fear/electric feeling in chest	Laughing	Vocalization, rotation to left and right, rapid paddling of bilateral legs, sitting up	–	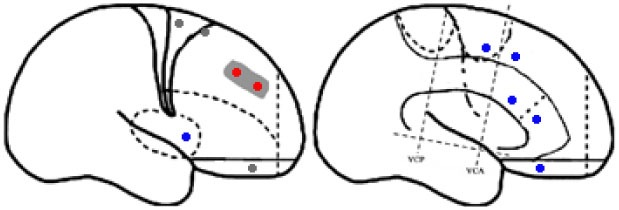	FCD IIb
6	MRI negative/R. ventromedial prefrontal cortex and temporal pole hypometabolism	Fear/fluster	Fearful expression	Rotation to left or right and trunk twisted, rapid pedaling of bilateral legs with large amplitude, vocalization, sitting and stand up	–	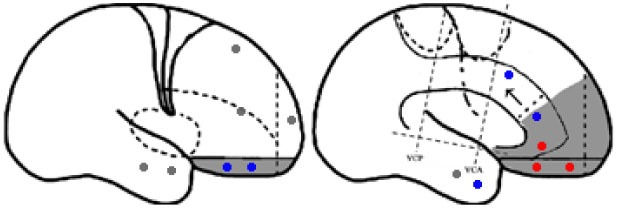	FCD Ia

*L, left; R, right; SEEG, stereo-electroencephalography; HMS, hypermotor seizures; FCD, focal cortical dysplasia*.

Dots in the schematic mean location of the SEEG electrodes and arrows refer to the spreading direction. Shaded areas refer to surgical resection site. (Red dots exhibited the electrodes with first clear ictal SEEG onset; blue dots refer to the spreading electrodes at the beginning of HMS onset.)

**Table 2:** Seizure semiology and surgery resection in patients with HMS2.

	Imaging characteristics (MRI/PET-MRI coregistration)	Aura	Affective components of HMS	Motor components of HMS	Tonic signs during HMS	Surgery resection	Histopathology
1	*L. insula*, operculum and anterior temporal lobe hyperintensity/*L. insula*, operculum and mesial temporal lobe hypometabolism	Uncomfortable feeling in right hand	–	Slight pedaling movements of lower limbs, sitting up	Bilateral arms and left legs	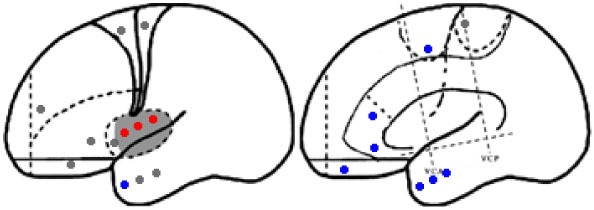	FCD I
2	MRI negative/L. IMC, pACC hypometabolism	None	–	Covering face with clothes, bilateral legs swinging, rotation to left, pelvic thrusting	–	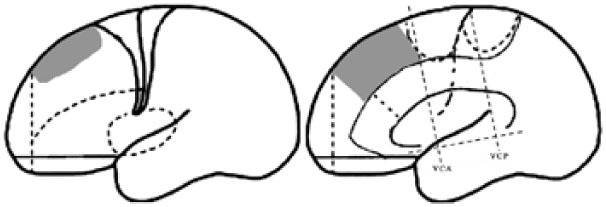	FCD IIa
3	MRI negative/IMC hypometabolism	Fear/fluster	–	Pouting, turn to one side (left or right), trunk twisting left and right, vocalization	Bilateral arms	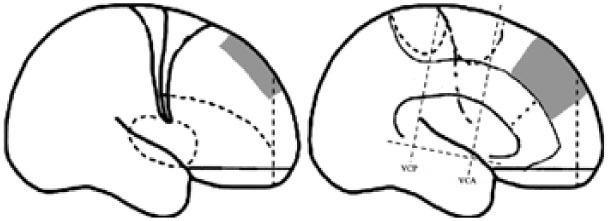	FCD IIa
4	Bottom of R. superior frontal sulcus abnormality/bottom of R. superior frontal sulcus hypometabolism	Blurred vision	–	Head-eye version to left, sitting up and body rock back and forth slowly, coughing, and vocalization	Sometimes bilateral arms	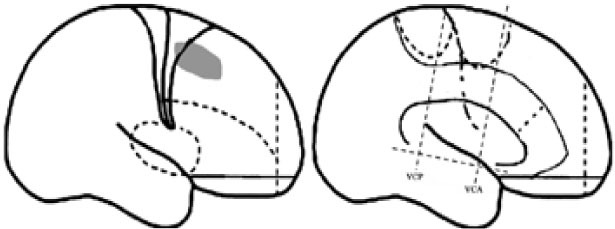	FCD IIb
5	L. temporal pole, mesial temporal structures and insular hyperintensity/L. temporal pole, mesial temporal structures and insula hypometabolism	Auditory hallucination/déjà vu/nausea/paresthesia pharynges/abnormal sensation in right body	Painful expression	Body rocking back and forth slowly, vocalization	Left arm tonic	NA	NA
6	L. inferior frontal sulcus hyperintensity and increased cortical thickness/L. inferior frontal sulcus and lateral OFC hypometabolism	None	–	Paddling of bilateral legs, rotation to left and lying on bed, upper trunk raising slowly up and down, pelvic thrusting	–	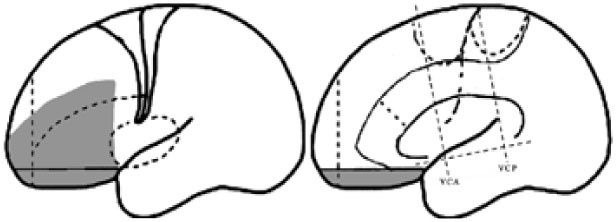	FCD IIb
7	L. hippocampal atrophy/L. temporal lobe hypometabolism	None	–	Sitting up and staring aimlessly, moving back and forth with mild agitation	–	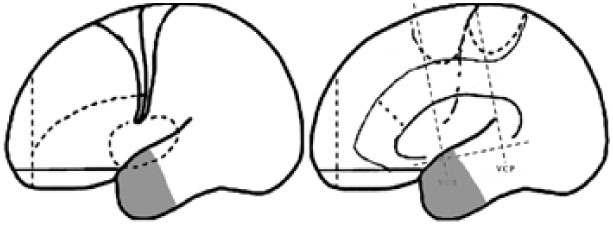	Gliosis
8	R. frontal operculum hyperintensity/R. central and frontal operculum hypometabolism	Fluster/nervous/paresthesia pharynges/laryngopharynx numbness	–	Cover mouth with left hand and then right hand, rotation to left and right, slight paddling of bilateral legs, climbing forward	–	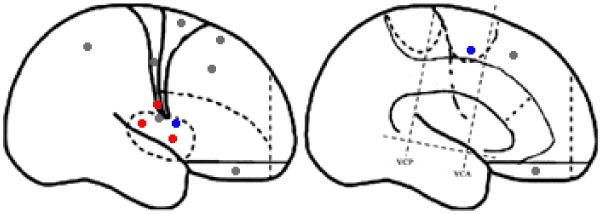	NA

*L, left; R, right; SEEG, stereo-electroencephalography; HMS, hypermotor seizures; OFC, orbitofrontal cortex; FCD, focal cortical dysplasia; pACC, pregenual anterior cingulate cortex*.

Dots in the schematic mean location of the SEEG electrodes. Shaded areas refer to surgical resection site. (Red dots exhibited the electrodes with first clear ictal SEEG onset; blue dots refer to the spreading electrodes at the beginning of HMS onset.)

### PET Findings

**Figure 2:**
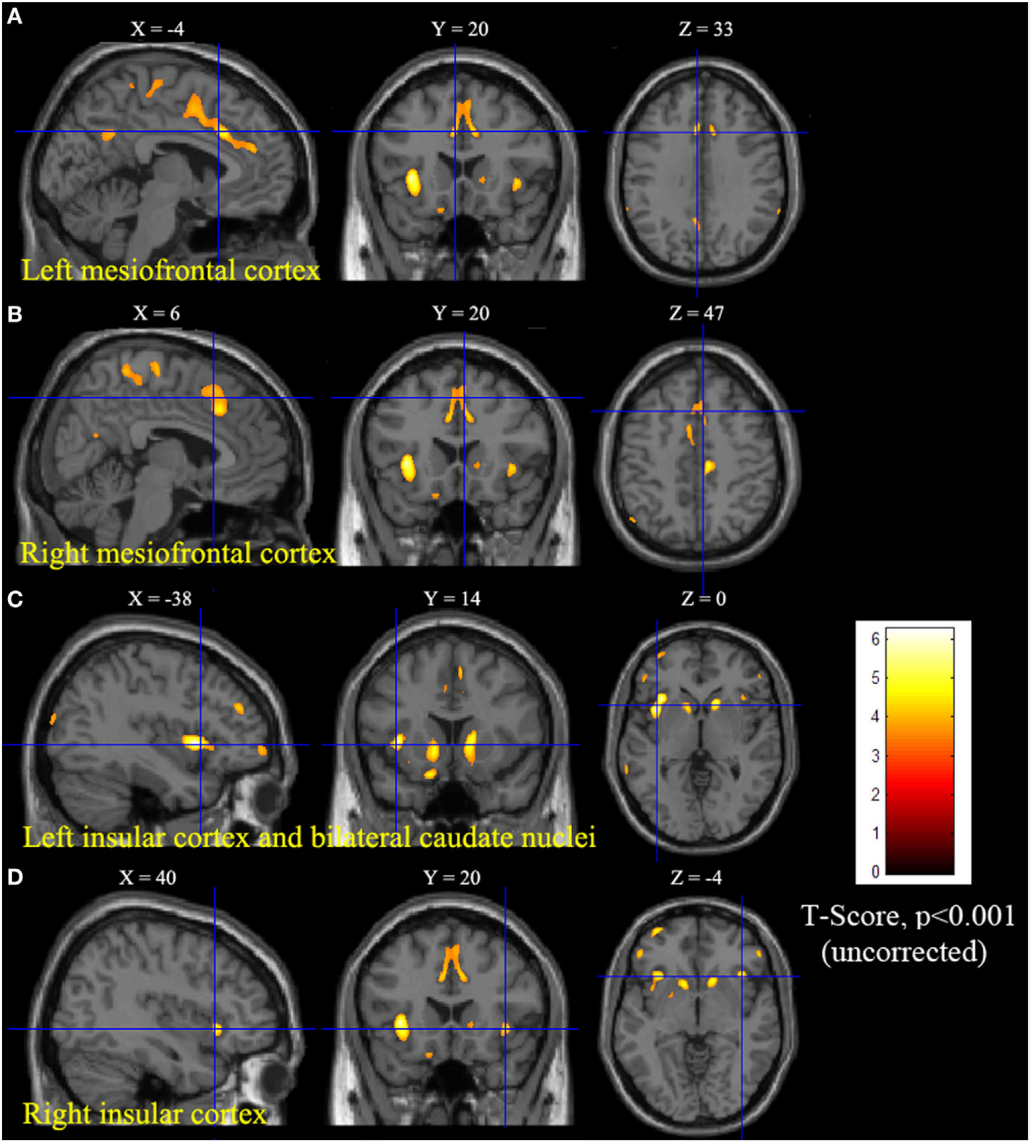
Statistical parametric mapping group analysis between patients with hypermotor seizures (HMS) (*n* = 14) and a control group (*n* = 18). The results showed significant hypometabolism in bilateral mesiofrontal cortex [**(A)** left pregenual anterior cingulate cortex, MCC, and mesial premotor cortex (MPMC); **(B)** right MPMC] and the bilateral anterosuperior insular lobes, the heads of the caudate nuclei [**(C)** for left and **(D)** for right] in patients with HMS. Note that PET images in patients with right-sided epileptogenic zones were transposed horizontally, and all HMS were supposed to originate from the left hemisphere. The color scale means T scores.

**Figure 3:**
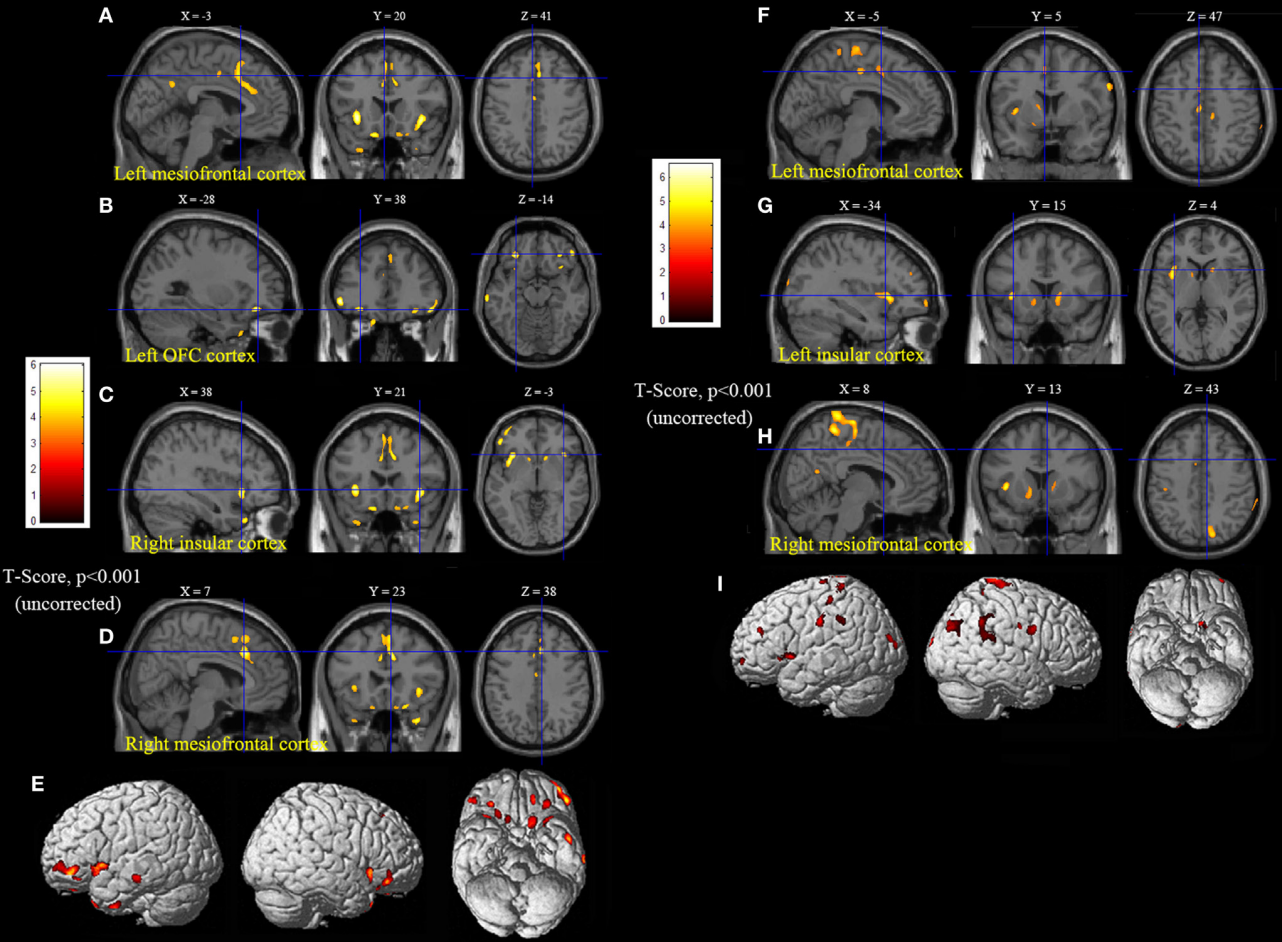
Statistical parametric mapping analysis results from the HMS1 group **(A–E)** and the HMS2 group **(F–I)** compared with the control group. The anterosuperior insular lobe, the head of the caudate nucleus, and the mesiofrontal cortex were found to be hypometabolic in each hypermotor seizures (HMS) subgroup. Compared with the HMS2 group, patients in the HMS1 group showed more extensive hypometabolic regions, including the left cingulate cortex **(A)**, left orbitofrontal cortex **(B,E)**, right insular cortex **(C)**, and left temporal pole **(E)**. Note that PET images in patients with right-sided epileptogenic zones were transposed horizontally, and all HMS were supposed to originate from left hemisphere. The color scale means T scores for the slice view. The surface view of significant results was presented in the 3D map **(E,I)** and color bar was not provided.

**Figure 4:**
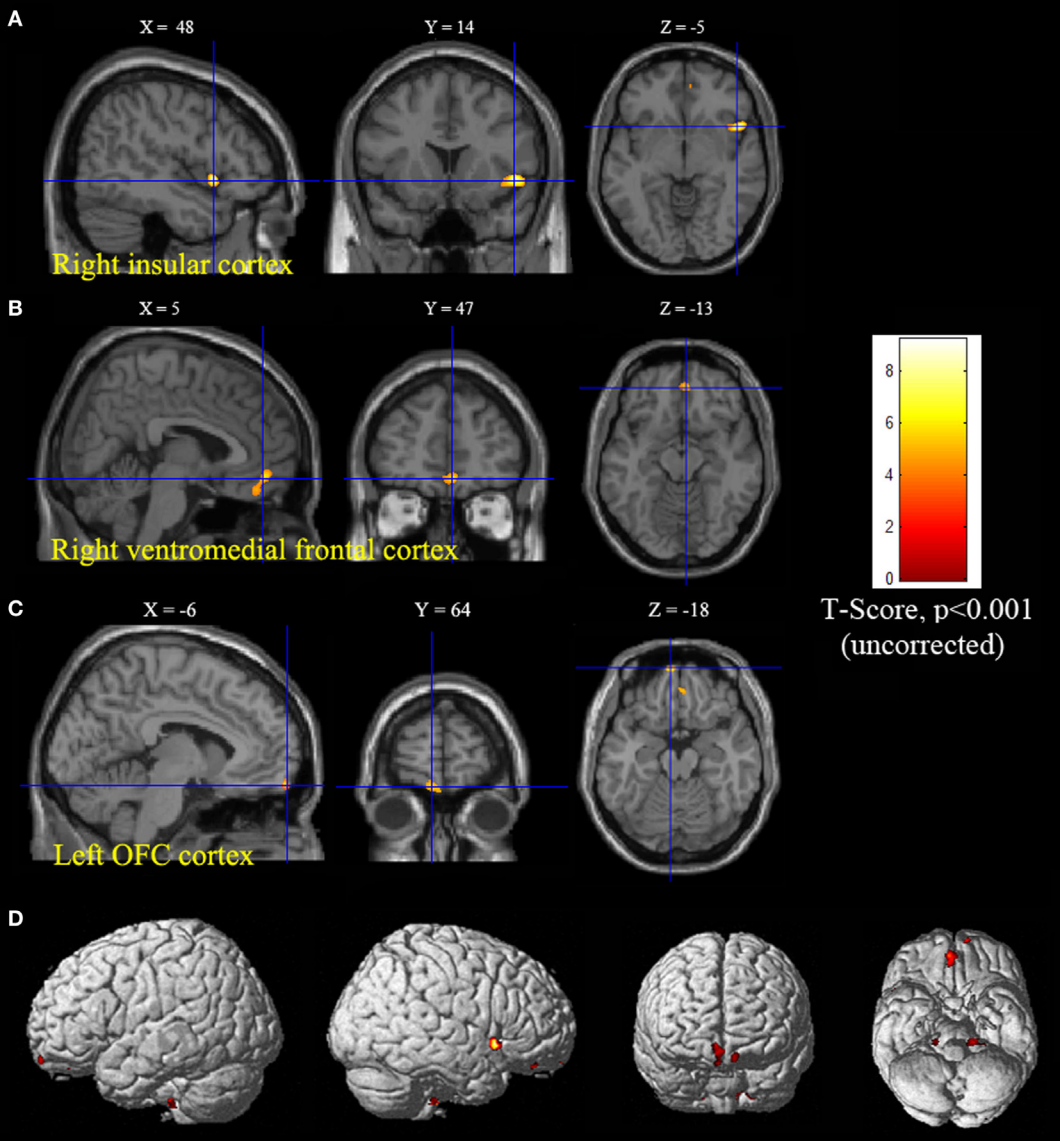
Significant hypometabolism in patients with HMS1, in comparison to patients with HMS2. More extensive hypometabolic areas in HMS1 than HMS2 were found, including right insular cortex **(A)**, right ventromedial frontal cortex **(B)**, and left orbitofrontal cortex **(C)**. Note that PET images in patients with right-sided epileptogenic zones were transposed horizontally, and all hypermotor seizures (HMS) were supposed to originate from left hemisphere. The color scale means T scores for the slice view. The superacial view of significant results was presented in the 3D map **(D)** and color bar was not provided.

Statistical parametric mapping analysis demonstrated hypometabolism in the bilateral anterosuperior insular lobes, bilateral mesial frontal lobes (MPMC, MCC, and pACC, left prominent), and the bilateral heads of the caudate nuclei in patients with HMS when compared with the control group (Figure 2; Table 3). Largely concordant results were achieved when comparing patients from the two HMS subgroups separately with controls. We further found significant hypometabolism in the bilateral OFC (left prominent) and left temporal pole in patients with HMS1, while no significant hypometabolism was found in these two areas nor in the right insula in HMS2 patients. In the mesiofrontal cortex, the hypometabolic area was more extensive in HMS1 patients than in HMS2 patients, particularly in the pACC and aMCC (Figure 3; Table 3). Besides, several extra cortical regions, including bilateral sensorimotor cortex, right supramarginal gyrus, parieto-occipital cortex, etc., were found to be significantly hypometabolic in patients with HMS2 (Figure 3; data were not shown in Table 3). When directly comparing PET imaging between patients with HMS1 and patients with HMS2, significant hypometabolism in patients with HMS1 was found in right insular cortex, right ventromedial prefrontal cortex, as well as left OFC, which also suggested that more extensive hypometabolic areas existed in patients with HMS1 (Figure 4; Table 3).

**Table 3:** Brain regions with significant hypometabolism in PET analysis.

Brain regions	Coordinates			Extent voxels (*N*)	*T* value	*Z*-score	*p*-Value (unc.)
	*X*	*Y*	*Z*				
**Whole HMS patients vs. control**							
*L. insula*	−38	11	1	2,146	6.27	4.97	<0.001
*R. insula*	39	22	−3	245	4.86	4.14	<0.001
Mesiofrontal cortex (bilateral)	−4	23	32	3,273	4.76	4.08	<0.001
L. caudate-putamen nucleus	−14	14	−21	2,586	4.72	4.05	<0.001
R. caudate-putamen nucleus	14	16	0	1,351	5.29	4.41	<0.001

**Patients with HMS1 vs. control**							
*L. insula*	−32	17	−8	1,473	6.00	4.57	<0.001
*R. insula*	38	21	−7	525	5.69	4.42	<0.001
Mesiofrontal cortex (bilateral)	7	24	37	3,367	5.58	4.36	<0.001
L. caudate	−10	14	−2	232	3.90	3.36	<0.001
R. caudate	14	16	1	335	4.57	3.79	<0.001
L. OFC (lateral)	−49	39	−6	1,080	5.89	4.51	<0.001
L. OFC (medial)	−13	17	−23	438	5.25	4.18	<0.001
L. temporal pole	36	24	−37	405	4.89	3.98	<0.001

**Patients with HMS2 vs. control**							
L. mesiofrontal cortex (ant. cingulate sulcus)	−5	3	49	109	3.83	3.35	<0.001
L. mesiofrontal cortex (post. cingulate sulcus)	−5	−17	47	188	4.42	3.74	<0.001
*L. insula*	−38	10	1	1,124	5.62	4.44	<0.001
L. caudate-putamen nucleus	−11	14	−3	670	4.05	3.50	<0.001
R. caudate-putamen nucleus	15	17	0	350	4.52	3.81	<0.001

**Patients with HMS1 vs. HMS2**							
R. insula	50	15	−5	623	8.36	4.6	<0.001
R. ventomedial prefrontal cortex	6	48	−12	492	5.39	3.69	<0.001
L. OFC (mesial)	−7	62	−16	317	5.88	3.88	<0.001

*Parts of significant hypometabolic areas were not shown in this table*.

*L, left; R, right; HMS, hypermotor seizures; OFC, orbitofrontal cortex*.

### SEEG Findings

**Figure 5:**
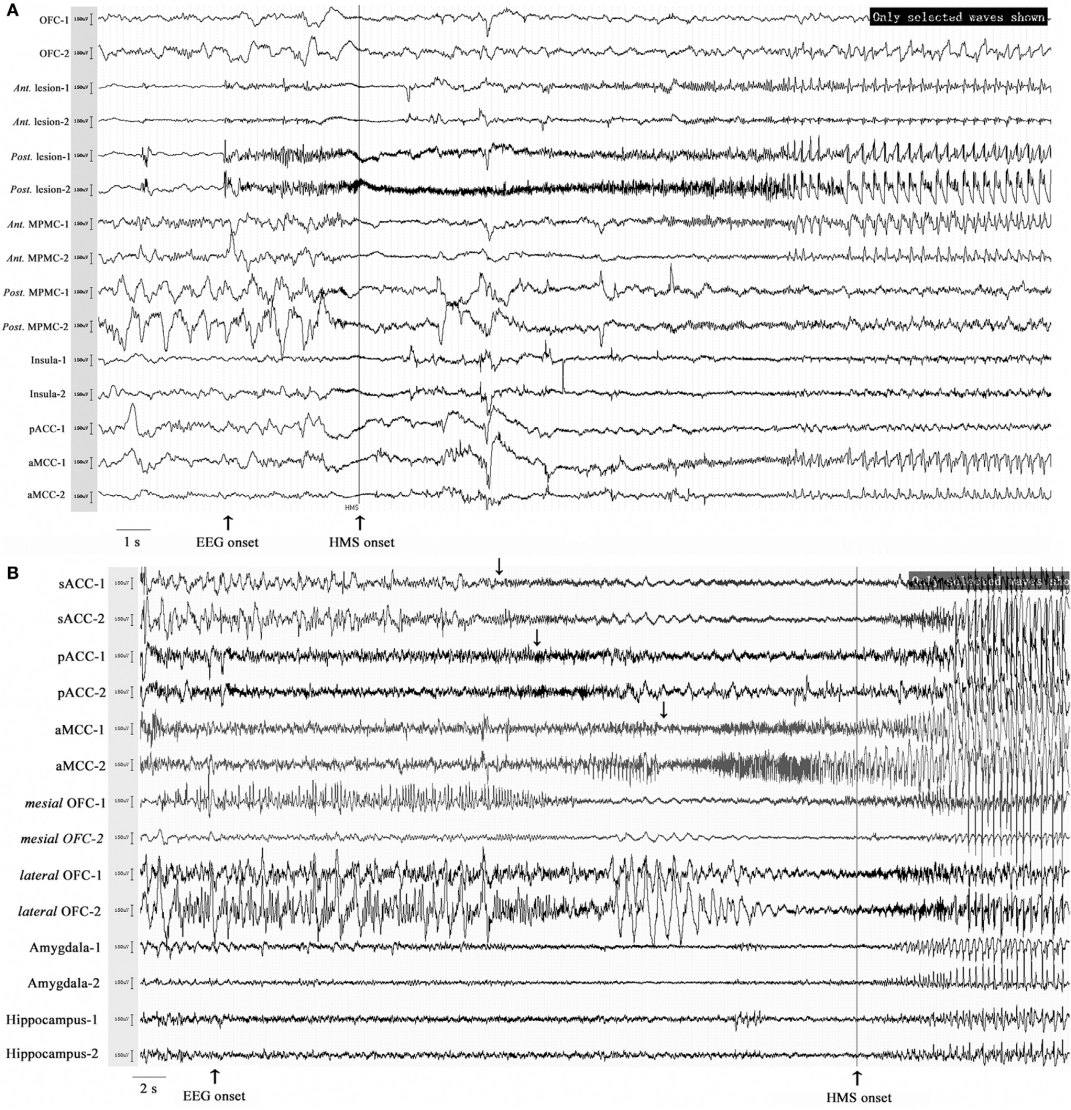
Stereo-electroencephalography recordings and hypermotor seizure (HMS) occurrence. In patient HMS1-5 **(A)**, ictal onset discharge originated from a dysplastic lesion in the anterior portion of the superior frontal sulcus, then spread to the mesial premotor cortex, insula, anterior cingulate cortex and MCC, and HMS occurred within a short time after electroencephalography (EEG) onset. In patient HMS1-6 **(B)**, orbitofrontal cortex (OFC) (contact: mesial OFC-1) was the epileptogenic focus. The ictal discharge spread along the subgenual anterior cingulate cortex (sACC)–pregenual anterior cingulate cortex (pACC)–anterior MCC (aMCC) (*black arrow*), and thereafter, HMS occurred clinically.

Six patients with HMS1 and two patients with HMS2 underwent SEEG electrode implantation. The cerebral areas predominately involved during the occurrence of HMS included the OFC (7/8), the ACC (6/6), the insula (5/6), the MCC (5/5), and the MPMC (4/4). Five patients underwent SEEG implantation in both the ACC and the MCC. Among these five patients, lateral frontal cortex (DLPMC, ILC)-originating epilepsy was found in two patients (HMS1-3 and HMS1-5) according to SEEG recordings and ictal onset discharge spread quickly into the MCC and ACC with HMS occurring shortly after EEG onset (Figure 5A). OFC-originating epilepsy was found in other three patients (pts. HMS1-1, 2, and 6); ictal onset discharge spread along the OFC–ACC–MCC pathway and HMS occurred clinically (Figure 5B).

## Discussion

In the present study, despite various epileptogenic zone locations, a specialized network was found with whole-brain voxel-based interictal PET analysis, including the anterosuperior insula, the mesiofrontal cortex (MCC-MPMC), and the caudate nucleus, which may contribute to the generation of HMS behavior. Patients with HMS1 has more extensive hypometabolic areas than HMS2, including OFC, pACC, and temporal pole, which may be related to the affective components in HMS.

In fact, our knowledge of HMS remains limited to independent cortical areas and no comprehensive symptomatic network has been studied. Our findings, which remain to be confirmed in larger studies, might help to have a better knowledge of HMS network and to optimize the placement of intracranial electrodes in patients with HMS. Similarly, previous clinical studies support our findings that HMS behavior could originate in the anterosuperior aspect of the insular lobe (4), ventromesial frontal cortex, or MPMC (2). Ictal SPECT analysis of HMS onset also found significant hyperperfusion in the mesiofrontal lobe, caudate nucleus and insular lobe (6). Previous studies have shown that interictal metabolic changes in PET imaging are correlated with ictal electroclinical patterns (9) or ictal brain SPECT perfusion (15), like ictal emotional/somesthetic symptoms and respectively interictal anterior/posterior insular hypometabolism (16), déjà-vu and interictal parahippocampal hypometabolism (7), or pouting and interictal ACC hypometabolism (17).

Mesiofrontal cortex was the common hypometabolic area both in HMS1 and HMS2. SEEG recordings also demonstrated that MCC or MPMC was one of the most frequently spreading areas at the beginning of HMS onset and further support the PET results. Histological studies of human brain demonstrated that a gigantopyramidal area existed in both banks of cingulate sulcus between the transverse plane levels of commissure anterior and corpora mamillaria (18), which is consistent with hand and foot representation area found in fMRI study of humans (19). The MCC has skeletomotor functions *via* projections to the spinal cord, striatum, MPMC, periaqueductal gray, and the pontine/cerebellar systems (12, 20). The aMCC is also responsible for fear-related motor behavior (13), and aMCC stimulation has been reported to evoke affective responses, including fear, with moderate or intense agitation (21). Thus, our arbitrary postulation is that the MCC and adjacent MPMC, especially bilateral banks of the cingulate sulcus, may play key roles in the symptomatic network of HMS.

Hyperkinetic seizures are one of the primary complex motor behaviors in insular lobe epilepsy (4, 22) and insular hypometabolism was also found in present patient group with HMS. The insular lobe has close connections with the mesial frontal lobe and HMS motor signs usually appear when ictal discharge spreads to mesiofrontal regions (cingulate cortex and supplementary motor area) in patients with insular epilepsy (22). Previous research has suggested that a higher-order synthesis of information from the amygdala, ACC, and OFC occurs in the anterior insula (23). The involvement of the anterior insula in HMS suggests that the insula might play a pivotal role in emotional and cognitive information processing in the limbic and paralimbic systems. Besides, the bilateral caudate nuclei were also involved in HMS generation, which is consistent with the results of a previous ictal SPECT study (6). Direct projections from the cingulate cortex to the dorsolateral striatum have been seen in primate studies (24, 25) and could contribute to the generation of motor behavior (4).

When comparing with health controls, patients with HMS1 demonstrated more extensive hypometabolic areas than HMS2, including pACC, aMCC, OFC, temporal pole. The pACC and aMCC have reciprocal connections with the amygdala (26), which has a pivotal role in emotion, particularly fear. The caudal terminal of the pACC innervates the facial nucleus and is responsible for facial expressions (20). The involvement of pACC and aMCC may account for the ictal signs of fear and/or neurovegetative symptoms and angry or fearful expressions commonly occurring in patients with HMS1. Interestingly, a previous, similar PET analysis (17) found that the ACC and the anterior portion of the insula were involved in ictal pouting. SEEG analysis demonstrated that pouting with intense facial expressions was related to rostroventral “affective” ACC activity, and less intense facial expressions were related with dorsal “cognitive” ACC activity. Although no significant hypometabolism in pACC and aMCC was found in direct comparison between HMS1 and HMS2, ventromedial prefrontal cortex was closely associated with emotion processing (27). In a study by Rheims et al. (2), HMS1 and HMS2 were associated with the ventromesial frontal cortex and the MPMC, respectively. Different types of HMS might be associated with different entry points into the HMS network. When the ictal onset zone is located in the ventromesial frontal lobe or the OFC, the discharge may spread across the sACC, pACC, and MCC, which is in accordance with the spreading direction detected by SEEG in patients with HMS1 in the present and previous studies (2); the clinical presentation may be characterized by intensive emotional patterns, neurovegetative signs, facial expressions, and HMS. When seizure discharge originates or spreads directly into the MCC and/or MPMC without ACC involvement, HMS may be presented with a less intense affective component.

Temporal pole is considered as the most possible epileptogenic zone in temporal lobe epilepsy related HMS (TLE-HMS). Patients with TLE-HMS have higher epileptogenicity in temporo-polar and OFC than TLE patients without HMS (28). In the present study, patients with HMS1 showed more significant hypometabolism in temporal pole and OFC than patients with HMS2 in the comparison with healthy control and direct comparison between two subgroups. Important and numerous bidirectional projections between these two regions have been confirmed in primate brain (29) and dysfunction of this focal network may contribute to the emotional changes during HMS1. Besides, amygdala is also closely connected with OFC and involved in the neural circuit underlying fear. The present study demonstrated no significant hypometabolism in amygdala. The question may be explained as follows: first, temporal pole contributes more to the generation of emotional changes than amygdala in HMS1. In the temporal lobe originated HMS, epileptogenicity of temporal pole and OFC were found to be higher than that of amygdala (28); second, although amygdala was involved in the HMS1, slight dysfunction did not influence the whole metabolic state of amygdala. The role of amygdala in the generation of HMS affective components needs further explored in the field of neurophysiological study.

Besides, several extra cortical regions, including bilateral sensorimotor cortex, right supramarginal gyrus, parieto-occipital cortex, etc., were found to be significantly hypometabolic in patients with HMS2 (Figure 3). Epileptogenic zones in these areas were seldom reported to contribute to the onset of HMS and thus were not discussed in detail the present study. Whether these hypometabolic regions were specific to HMS remains to be determined. In the results of SPECT study, hyperfusion was found in bilateral parasagittal sensorimotor areas during HMS onset (6). More accompanied seizure semiology, including tonic or clonic motor behavior, may suggest the involvement of peri-sensorimotor areas.

The main limitation of this study remains the weak causality between the interictal hypometabolic network and HMS behavior, because clinical signs evolve as the epileptic discharge spreads in both time and space (30). Although we recorded the cortical regions recruited at the occurrence of HMS by SEEG, the period of HMS production occurs with variable time lags after discharge. Not all areas with interictal hypometabolism (1) or epileptic discharges during HMS were the cause of hyperkinetic behavior. The second major limitation refers to the PET analysis results achieved under *p* < 0.001 without correction for multiple comparisons. Additional limitations are the limited number of patients included and the lack of a second control group (epilepsy patients without HMS). In the SPM analysis, we flipped the PET images horizontally in patients with a right-sided epileptogenic zone, however, brain networks may be different between right and left hemispheres. Besides, patient selection bias may exist because only patients with PET scans were included in the analysis.

## Conclusion

We have identified a common interictal hypometabolic pattern in patients with HMS. A network involving the anterosuperior insula, mesiofrontal cortex (MCC-MPMC), and caudate nucleus may contribute to the generation of HMS. ACC, OFC, and temporal pole are possibly associated with the affective components of HMS. Our findings provide further insight into understanding the network basis of HMS semiology.

## Ethics Statement

Informed consent with protocols approved by the Institutional Review Boards of the Beijing Tiantan Hospital were obtained from all included members. The study has been performed in accordance with the ethical standards laid down in the 1964 Declaration of Helsinki and its later amendments.

## Author Contributions

XW: acquisition of data, statistical analysis, and drafting the manuscript; WH, XS, and KZ: acquisition and interpretation of data, revising the manuscript for intellectual content; YM, LS, and CZ: acquisition of data and revising the manuscript for intellectual content; ZZ and JL: acquisition and interpretation of data; J-gZ: study design, study supervision, and final revising the manuscript for intellectual content.

## Conflict of Interest Statement

The authors declare that the research was conducted in the absence of any commercial or financial relationships that could be construed as a potential conflict of interest.
